# PREMIGRATION, DISPLACEMENT, AND POSTMIGRATION EXPERIENCES OF LONELINESS AMONG HMONG OLDER ADULTS

**DOI:** 10.1093/geroni/igz038.3292

**Published:** 2019-11-08

**Authors:** Cindy Vang, Michael Sieng

**Affiliations:** 1 University of Minnesota-Twin Cities School of Social Work, Saint Paul, Minnesota, United States; 2 Arizona State University, Tempe, Arizona, United States

## Abstract

Available evidence highlights the detrimental impact of loneliness on the mental and physiological health of older adults. While immigrant older adults report higher prevalence for experiencing loneliness compared to native born older adults, minimal research has simultaneously explored the premigration, displacement, and postmigration experiences of loneliness among older adults with a refugee history. This study aimed to explore loneliness in these three phases among Hmong older adults, an ethnic minority group resettled in the United States as refugees over 40 years ago. Drawing on data from a constructivist grounded theory study guided by an intersectionality framework, the first author interviewed 17 community-dwelling Hmong older adults age 65 and older residing in Fresno and Sacramento, California. Two coders coded and analyzed the transcribed interviews. Findings revealed negative, disruptive, and discriminatory experiences underscored by systems of oppression grounded on the social, political, psychological, and cultural context of each phase. Influencing factors that contributed to loneliness were identified as: trust, loss, aging-related issues, isolation, sense of community, access to cultural community, instability, violence, and cultural adjustments. In specific phases, particular influencing factors were more evident and persistent in producing loneliness. Some influencing factors remained a problem for participants across all phases. With the unprecedented growth of refugees all over the world, this study highlights the need for more research, practice, and policy focused on the context of the refugee experience to gain greater insight into their loneliness experiences and expand the notion of loneliness as an individual experience.

